# Supplementary material for: Gender, skin color, and household composition explain inequities in household food insecurity in Brazil
Source: PLOS Glob Public Health. 2023 Oct 3;3(10):e0002324. doi: 10.1371/journal.pgph.0002324 (PMC10547153; doi:10.1371/journal.pgph.0002324)
Supplement: S1 Table — FI: Food insecurity. 1PNAD (Pesquisas Nacionais por Amostras de Domicílios): Brazilian National Households Sample Surveys; 2POF (Pesquisa de Orçamentos Familiares): Household Budget Survey. (DOCX) [file pgph.0002324.s001.docx]

**S1 Table. Prevalence (%) of moderate/severe food insecurity by gender of reference person of the household in national level and in the different macroregions of Brazil (2004, 2013 and 2018).**

| Macroregion | Sex | PNAD^1^ 2004 | | PNAD^1^ 2013 | | POF^2^ 2018 | |
| --- | --- | --- | --- | --- | --- | --- | --- |
|  |  | **Moderate/severe FI (%)** | **(95% IC)** | **Moderate/severe FI (%)** | **(95% IC)** | **Moderate/severe FI (%)** | **(95% IC)** |
| National | Men | 15.5 | (15.0-16.1) | 7.2 | (6.9-7.4) | 10.8 | (10.3-11.3) |
| National | Women | 21.1 | (20.5-21.8) | 9.2 | (8.8-9.5) | 15.3 | (14.7-15.3) |
| North | Men | 23.4 | (21.3-25.7) | 14 | (12.8-15.1) | 24.3 | (22.2-26.7) |
| North | Women | 31.5 | (29.2-33.9) | 16 | (14.6-17.4) | 26.3 | (24.1-28.6) |
| Northeast | Men | 30.1 | (28.3-32.0) | 13.8 | (13.0-14.6) | 18.3 | (17.2-19.4) |
| Northeast | Women | 33.2 | (31.6-34.8) | 15.9 | (15.0-16.9) | 23.3 | (22.0-24.5) |
| Central-West | Men | 11.6 | (10.5-12.7) | 5.0 | (4.4-5.8) | 10.6 | (9.3-12.1) |
| Central-West | Women | 18.2 | (16.5-19.9) | 6.3 | (5.5-7.3) | 14.0 | (12.3-16.0) |
| Southeast | Men | 9.2 | (8.7-9.7) | 3.7 | (3.3-4.0) | 6.9 | (6.2-7.6) |
| Southeast | Women | 15.5 | (14.6-16.4) | 5.7 | (5.2-6.1) | 11.3 | (10.1-12.5) |
| South | Men | 8.0 | (7.4-8.6) | 3.7 | (3.3-4.2) | 3.8 | (3.2-4.6) |
| South | Women | 13.9 | (12.6-15.2) | 5.5 | (4.9-6.2) | 7.5 | (6.3-8.8) |

¹PNAD (*Pesquisas Nacionais por Amostras de Domicílios*): Brazilian National Households Sample Surveys; ^2^POF (*Pesquisa de Orçamentos Familiares*): Household Budget Survey. FI: Food insecurity.
