# Supplementary material for: Gender, skin color, and household composition explain inequities in household food insecurity in Brazil
Source: PLOS Glob Public Health. 2023 Oct 3;3(10):e0002324. doi: 10.1371/journal.pgph.0002324 (PMC10547153; doi:10.1371/journal.pgph.0002324)
Supplement: S2 Table — *Statistical significance (p<0.05). Multinomial Logistic regression. Model adjusted for area of the household, presence of children under 5 years old, resident’s number and income. aRRR: adjusted relative risk ratio. 1PNAD (Pesquisas Nacionais por Amostras de Domicílios): Brazilian National Households Sample Surveys; 2POF (Pesquisa de Orçamentos Familiares): Household Budget Survey. (DOCX) [file pgph.0002324.s002.docx]

| **S2 Table.** **Logistic Models for relationship between moderate/severe Food Insecurity and profiles of reference person of households with sex, race and marital status stratified by region. Brazil, 2004, 2013 and 2018.** | | | | | | | | | | | | | | |
| --- | --- | --- | --- | --- | --- | --- | --- | --- | --- | --- | --- | --- | --- | --- |
| **Profiles** | | **PNAD¹ 2004 (N=117,731)** | | | | | | | | | | | | |
|  |  | **Brazil** | | | **North** | | | **Northeast** | | **Central-West** | | **Southeast** | | **South** |
|  |  | **aRRR (CI 95%)** | | | **aRRR (CI 95%)** | | | **aRRR (CI 95%)** | | **aRRR (CI 95%)** | | **aRRR (CI 95%)** | | **aRRR (CI 95%)** |
| Men, White, Married | | 1.0 | | | 1.0 | | | 1.0 | | 1.0 | | 1.0 | | 1.0 |
| Men, White, Single | | 1.99(1.74-2.28) | | | 1.48(0.79-2.78) | | | 1.46(1.12-1.88)* | | 1.16(0.77-1.76) | | 1.65(1.32-2.07)* | | 2.66(2.01-3.52)* |
| Women, White, Married | | 1.95(1.66-2.29) | | | 1.06(0.65-1.73) | | | 1.35(1.04-1.76)* | | 1.23(0.74-2.02) | | 2.45(1.83-3.27)* | | 1.93(1.41-2.64)* |
| Women, White, Single | | 2.05(1.89-2.22)* | | | 2.04(1.58-2.64)* | | | 1.39(1.20-1.59)* | | 1.85(1.45-2.35)* | | 1.94(1.69-2.24)* | | 2.48(2.09-2.95)* |
| Men, Black/Brown, Married | | 1.96(1.83-2.09)* | | | 1.63(1.37-1.95)* | | | 1.54(1.36-1.74)* | | 1.60(1.33-1.92)* | | 1.63(1.46-1.82)* | | 2.12(1.75-2.56)* |
| Men, Black/Brown, Single | | 3.46(3.09-3.88)* | | | 3.26(2.35-4.52)* | | | 2.55(2.12-3.07)* | | 2.70(1.99-3.65)* | | 2.48(1.96-3.13)* | | 3.59(2.48-5.19)* |
| Women, Black/Brown, Married | | 3.45(3.02-3.93)* | | | 2.99(2.17-4.11)* | | | 2.04(1.66-2.51)* | | 2.87(2.05-4.03)* | | 3.75(2.87-4.91)* | | 4.33(2.65-7.07)* |
| Women, Black/Brown, Single | | 3.78(3.48-4.10)* | | | 3.36(2.65-4.25)* | | | 2.68(2.38-3.08)* | | 2.67(2.21-3.23)* | | 3.44(2.99-3.95)* | | 4.29(3.16-5.83)* |
| **Profiles** | | **PNAD¹ 2013 (N=115,096)** | | | | | | | | | | | | |
|  |  | **Brazil** | | | **North** | | | **Northeast** | | **Central-West** | | **Southeast** | | **South** |
|  |  | **aRRR (CI 95%)** | | | **aRRR (CI 95%)** | | | **aRRR (CI 95%)** | | **aRRR (CI 95%)** | | **aRRR (CI 95%)** | | **aRRR (CI 95%)** |
| Men, White, Married | | 1.0 | | | 1.0 | | | 1.0 | | 1.0 | | 1.0 | | 1.0 |
| Men, White, Single | | 1.89(1.61-2.24) | | | 1.46(0.97-2.21) | | | 1.53(1.16-2.03)* | | 1.76(1.04-2.99)* | | 1.88(1.30-2.70)* | | 2.13(1.59-2.85)* |
| Women, White, Married | | 1.42(1.23-1.64) | | | 1.75(1.21-2.52)* | | | 1.35(1.06-1.72)* | | 0.69(0.39-1.22) | | 1.31(0.99-1.75) | | 1.61(1.23-2.10)* |
| Women, White, Single | | 2.02(1.79-2.29)* | | | 1.82(1.29-2.56)* | | | 1.56(1.28-1.90)* | | 1.48(1.03-2.12)* | | 2.13(1.62-2.79)* | | 2.39(1.90-3.02)* |
| Men, Black/Brown, Married | | 2.10(1.91-2.31)* | | | 1.87(1.49-2.33)* | | | 1.64(1.43-1.87)* | | 1.50(1.14-1.96)* | | 1.99(1.56-2.55)* | | 1.87(1.53-2.29)* |
| Men, Black/Brown, Single | | 3.36(2.96-3.81)* | | | 2.28(1.70-3.07)* | | | 2.45(2.05-2.94)* | | 2.40(1.65-3.51)* | | 2.64(1.80-3.87)* | | 3.56(2.76-4.60)* |
| Women, Black/Brown, Married | | 2.54(2.26-2.86)* | | | 2.09(1.60-2.75)* | | | 1.89(1.61-2.23)* | | 1.42(0.99-2.02) | | 2.21(1.55-3.14)* | | 2.40(1.85-3.11)* |
| Women, Black/Brown, Single | | 3.57(3.21-3.98)* | | | 2.73(2.14-3.48)* | | | 2.64(2.25-3.10)* | | 2.66(1.92-3.69)* | | 3.98(2.90-5.47)* | | 3.54(2.87-4.38)* |
| **Profiles** | **POF² 2018 (N=57,204)** | | | | | | | | | | | | | |
|  |  | | | **Brazil** | | **North** | | | **Northeast** | | **Central-West** | | **Southeast** | **South** |
|  |  |  |  | **aRRR (CI 95%)** | | **aRRR (CI 95%)** | | | **aRRR (CI 95%)** | | **aRRR (CI 95%)** | | **aRRR (CI 95%)** | **aRRR (CI 95%)** |
| Men, White, Married | | | 1.0 | | | | 1.0 | | 1.0 | | 1.0 | | 1.0 | 1.0 |
| Men, White, Single | | | 1.72(1.34-2.20) | | | | 1.61(0.81-3.24) | | 1.94(1.36-2.77)* | | 2.60(1.30-5.23)* | | 1.44(0.91-2.27) | 1.88(1.05-3.36)* |
| Women, White, Married | | | 1.46(1.20-1.77) | | | | 0.98(0.56-1.71) | | 1.49(1.12-1.99)* | | 2.07(0.99-4.31) | | 1.52(1.04-2.22)8 | 1.56(0.97-2.51) |
| Women, White, Single | | | 2.74(2.32-3.24) | | | | 1.10(0.60-1.99) | | 2.11(1.66-2.68)* | | 2.37(1.48-3.78)* | | 2.70(2.00-3.64)* | 4.05(2.76-5.95)* |
| Men, Black/Brown, Married | | | 2.12(1.86-2.41)* | | | | 1.26(0.91-1.73) | | 1.49(1.24-1.78)* | | 2.27(1.54-3.35)* | | 1.60(1.23-2.09)* | 2.11(1.33-3.35)* |
| Men, Black/Brown, Single | | | 3.83(3.25-4.52)* | | | | 2.22(1.42-3.48)* | | 2.47(1.94-3.14)* | | 3.80(2.35-6.15)* | | 3.25(2.31-4.52)* | 3.56(1.98-6.38)* |
| Women, Black/Brown, Married | | | 2.92(2.51-3.39)* | | | | 1.29(0.88-1.89) | | 2.03(1.64-2.51)* | | 1.92(1.23-3.00)* | | 2.50(1.79-3.50)* | 3.84(2.25-6.56)* |
| Women, Black/Brown, Single | | | 4.48(3.89-5.16)* | | | | 2.11(1.43-3.10)* | | 2.89(2.35-3.55)* | | 4.02(2.69-6.02)* | | 3.91(2.96-5.17)* | 5.89(3.66-9.48)* |
| *Statistical significance (p<0.05). Multinomial Logistic regression. Model adjusted for area of the household, presence of children under 5 years old, resident’s number and income. aRRR: adjusted relative risk ratio. ¹PNAD (*Pesquisas Nacionais por Amostras de Domicílios*): Brazilian National Households Sample Surveys; ^2^POF (*Pesquisa de Orçamentos Familiares*): Household Budget Survey. | | | | | | | | | | | | | | |
