# Supplementary material for: Gender, skin color, and household composition explain inequities in household food insecurity in Brazil
Source: PLOS Glob Public Health. 2023 Oct 3;3(10):e0002324. doi: 10.1371/journal.pgph.0002324 (PMC10547153; doi:10.1371/journal.pgph.0002324)
Supplement: S3 Table — Brazil, 2004, 2013 and 2018. FS: Foos Security; FI: Food Insecurity. 1PNAD (Pesquisas Nacionais por Amostras de Domicílios): Brazilian National Households Sample Surveys; 2POF (Pesquisa de Orçamentos Familiares): Household Budget Survey. (DOCX) [file pgph.0002324.s003.docx]

**S3 Table. Prevalence (%) of Food Security/Food Insecurity status of each profile of sex, race/skin color and marital status of the reference person of the household. Brazil, 2004, 2013 and 2018.**

| Profile/Food Insecurity Status | PNAD¹ 2004 | | | | | | |  | |
| --- | --- | --- | --- | --- | --- | --- | --- | --- | --- |
|  | **FS** | **95% CI** | **Mild FI** | | **95% CI** | | **Moderate/ Severe FI** | **95% CI** | |
| Men, White, Married | 76.2 | (75.5-76.9) | 14.7 | | (14.1-15.2) | | 9.1 | (8.7-9.5) | |
| Men, White, Single | 80.4 | (79.0-81.8) | 9.2 | | (8.2-10.3) | | 10.3 | (9.3-11.4) | |
| Women, White, Married | 68.0 | (65.7-70.2) | 19.0 | | (17.2-20.9) | | 13.0 | (11.5-14.6) | |
| Women, White, Single | 71.2 | (70.2-72.3) | 15.2 | | (14.4-15.9) | | 13.6 | (12.8-14.3) | |
| Men, Black/Brown, Married | 53.4 | (52.3-54.5) | 23.4 | | (22.6-24.2) | | 23.2 | (22.2-24.2) | |
| Men, Black/Brown, Single | 64.5 | (62.8-66.2) | 13.8 | | (12.7-15.0) | | 21.7 | (20.3-23.1) | |
| Women, Black/Brown, Married | 44.8 | (42.6-47.0) | 26.3 | | (24.5-28.3) | | 28.8 | (26.9-30.9) | |
| Women, Black/Brown, Single | 47.1 | (45.8-48.4) | 22.6 | | (21.6-23.6) | | 30.3 | (29.1-31.4) | |
| Profile/Food Insecurity Status | **PNAD¹ 2013** | | | | | | |  | |
|  | **FS** | **95% CI** | **Mild FI** | **95% CI** | | **Moderate/ Severe FI** | | | **95% CI** |
| Men, White, Married | 86.1 | (85.5-86.7) | 10.5 | (9.9-11.0) | | 3.4 | | | (3.1-3.7) |
| Men, White, Single | 87.5 | (86.4-88.4) | 7.8 | (7.1-8.7) | | 4.7 | | | (4.1-5.4) |
| Women, White, Married | 84.5 | (83.5-85.5) | 11.4 | (10.6-12.3) | | 4.0 | | | (3.6-4.5) |
| Women, White, Single | 83.0 | (82.1-83.9) | 11.0 | (10.3-11.8) | | 5.9 | | | (5.4-6.4) |
| Men, Black/Brown, Married | 70.5 | (69.7-71.4) | 19.5 | (18.8-20.2) | | 10.0 | | | (9.5-10.4) |
| Men, Black/Brown, Single | 74.8 | (73.7-76.0) | 13.7 | (12.9-14.7) | | 11.4 | | | (10.6-12.3) |
| Women, Black/Brown, Married | 67.9 | (66.7-69.1) | 21.0 | (20.0-22.0) | | 11.1 | | | (10.3-12.0) |
| Women, Black/Brown, Single | 66.6 | (65.5-67.6) | 19.6 | (18.8-20.4) | | 13.8 | | | (13.1-14.6) |
| Profile/Food Insecurity Status | **POF^2^ 2018** | | | | | | |  | |
|  | **FS** | **95% CI** | **Mild FI** | **95% CI** | | **Moderate/ Severe FI** | | | **95% CI** |
| Men, White, Married | 76.1 | (74.8-77.4) | 18.3 | (17.2-19.5) | | 5.6 | | | (5.0-6.2) |
| Men, White, Single | 80.0 | (77.7-82.1) | 12.5 | (10.8-14.4) | | 7.5 | | | (6.3-8.9) |
| Women, White, Married | 70.2 | (68.0-72.4) | 22.1 | (20.1-24.2) | | 7.6 | | | (6.5-8.9) |
| Women, White, Single | 69.5 | (67.7-71.3) | 19.6 | (18.2-21.2) | | 10.8 | | | (9.7-12.0) |
| Men, Black/Brown, Married | 57.4 | (56.0-58.7) | 28.5 | (27.4-29.6) | | 14.1 | | | (13.4-15.0) |
| Men, Black/Brown, Single | 61.6 | (59.4-63.7) | 20.7 | (19.0-22.6) | | 17.7 | | | (16.2-19.3) |
| Women, Black/Brown, Married | 48.9 | (47.0-50.8) | 32.8 | (31.1-34.6) | | 18.3 | | | (17.0-19.7) |
| Women, Black/Brown, Single | 50.0 | (48.3-51.4) | 29.4 | (28.1-30.8) | | 20.7 | | | (19.5-22.0) |

FS: Food Security; FI: Food Insecurity. ¹PNAD (*Pesquisas Nacionais por Amostras de Domicílios*): Brazilian National Households Sample Surveys; ^2^POF (*Pesquisa de Orçamentos Familiares*): Household Budget Survey.
