# Supplementary material for: Gender, skin color, and household composition explain inequities in household food insecurity in Brazil
Source: PLOS Glob Public Health. 2023 Oct 3;3(10):e0002324. doi: 10.1371/journal.pgph.0002324 (PMC10547153; doi:10.1371/journal.pgph.0002324)
Supplement: S4 Table — Brazil, 2004, 2013 and 2018. *Statistical significance (p<0.05). Multinomial Logistic regression. Models adjusted for region, area of the household, resident’s number and income. cRRR: crude estimates of relative risk ratio. 1PNAD (Pesquisas Nacionais por Amostras de Domicílios): Brazilian National Households Sample Surveys; 2POF (Pesquisa de Orçamentos Familiares): Household Budget Survey. (DOCX) [file pgph.0002324.s004.docx]

**S4 Table. Multinomial Logistic Models crude estimates for relationship between Food Security and Food Insecurity levels and profiles of reference person of households with sex, race and marital status stratified by presence of children under 5-years old on the household. Brazil, 2004, 2013 and 2018.**

| **PNAD¹ 2004** | | | | | | | | |
| --- | --- | --- | --- | --- | --- | --- | --- | --- |
|  | **Presence of children under 5 years old** | | | | **Absence of children under 5 years old** | | | |
|  | | | | | | | | |
| **Profiles** | **Mild FI** | **Moderate/severe FI** | | | **Mild FI** | | | **Moderate/severe FI** |
|  | **cRRR**  **(CI 95%)** | **cRRR**  **(CI 95%)** | | | **cRRR**  **(CI 95%)** | | | **cRRR**  **(CI 95%)** |
| Men, White, Married | 1.0 | 1.0 | | | 1.0 | | | 1.0 |
| Men, White, Single | 1.11 (0.71-1.76) | 2.15 (1.36-3.38)* | | | 0.69 (0.61-0.79)* | | | 1.22 (1.09-1.38)* |
| Women, White, Married | 1.41 (1.15-1.74)* | 1.49 (1.18-1.88)* | | | 1.43 (1.22-1.68)* | | | 1.63 (1.37-1.95)* |
| Women, White, Single | 1.73 (1.50-1.99)* | 2.66 (2.30-3.09)* | | | 1.17 (1.08-1.26)* | | | 1.63 (1.50-1.77)* |
| Men, Black/Brown, Married | 2.14 (1.97-2.32)* | 3.49 (3.19-3.82)* | | | 2.19 (2.05-2.34)* | | | 3.44 (3.21-3.69)* |
| Men, Black/Brown, Single | 2.28 (1.61-3.22)* | 5.46 (3.90-7.62)* | | | 1.26 (1.13-1.41)* | | | 3.13 (2.83-3.47)* |
| Women, Black/Brown, Married | 2.70 (2.27-3.22)* | 5.06 (4.25-6.02)* | | | 3.02 (2.64-3.47)* | | | 5.16 (4.47-5.94)* |
| Women, Black/Brown, Single | 3.08 (2.69-3.52)* | 6.88 (6.04-7.83)* | | | 2.55 (2.36-2.75)* | | | 6.37 (4.96-5.81)* |
| **PNAD¹ 2013** | | | | | | | | |
|  | **Presence of children under 5 years old** | | | **Absence of children under 5 years old** | | | | |
| **Profiles** | **Mild FI** | **Moderate/severe FI** | | **Mild FI** | | | **Moderate/severe FI** | |
|  | **cRRR**  **(CI 95%)** | **cRRR**  **(CI 95%)** | | **cRRR**  **(CI 95%)** | | **cRRR**  **(CI 95%)** | | |
| Men, White, Married | 1.0 | 1.0 | | 1.0 | | 1.0 | | |
| Men, White, Single | 1.36 (0.96-1.92) | 2.24 (1.40-3.60)* | | 0.84 (0.75-0.96)* | | 1.55 (1.31-1.83)* | | |
| Women, White, Married | 1.13 (0.97-1.33) | 1.53 (1.19-1.98)* | | 1.17 (1.05-1.30)* | | 1.34 (1.14-1.57)* | | |
| Women, White, Single | 1.38 (1.16-1.65)* | 2.82 (2.19-3.63)* | | 1.16 (1.06-1.28)* | | 1.80 (1.58-2.05)* | | |
| Men, Black/Brown, Married | 1.94 (1.76-2.15)* | 3.64 (3.11-4.28)* | | 2.20 (2.06-2.37)* | | 3.32 (2.98-3.69)* | | |
| Men, Black/Brown, Single | 2.37 (1.86-3.02)* | 5.37 (4.04-7.14)* | | 1.56 (1.41-1.72)* | | 3.68 (3.23-4.18)* | | |
| Women, Black/Brown, Married | 1.97 (1.75-2.23)* | 3.51 (2.90-4.26)* | | 2.48 (2.26-2.72)* | | 3.86 (3.38-4.40)* | | |
| Women, Black/Brown, Single | 2.75 (2.41-3.13)* | 7.24 (6.03-8.70)* | | 2.41 (2.22-2.62)* | | 4.78 (4.26-5.36)* | | |
|  |  |  | |  | |  | | |
| **POF² 2018** | | | | | | | | |
|  | **Presence of children under 5 years old** | | **Absence of children under 5 years old** | | | | | |
| **Profiles** | **Mild FI** | **Moderate/severe FI** | | **Mild FI** | | | **Moderate/severe FI** | |
|  | **cRRR**  **(CI 95%)** | **cRRR**  **(CI 95%)** | | **cRRR**  **(CI 95%)** | | **cRRR**  **(CI 95%)** | | |
| Men, White, Married | 1.0 | 1.0 | | 1.0 | | 1.0 | | |
| Men, White, Single | 0.41 (0.17-0.98)* | 1.15 (0.42-3.10) | | 0.74 (0.61-0.89)* | | 1.35 (1.08-1.70)* | | |
| Women, White, Married | 1.02 (0.76-1.36) | 1.26 (0.78-2.04) | | 1.40 (1.19-1.64)* | | 1.53 (1.24-1.88) | | |
| Women, White, Single | 1.86 (1.34-2.57)* | 2.84 (1.83-4.42)* | | 1.22 (1.06-1.39)* | | 2.16 (1.82-2.56)* | | |
| Men, Black/Brown, Married | 1.86 (1.55-2.25)* | 3.28 (2.47-4.37)* | | 2.01 (1.80-2.24)* | | 3.24 (2.81-3.72)* | | |
| Men, Black/Brown, Single | 1.86 (1.15-3.02)* | 4.22 (2.20-8.10)* | | 1.52 (1.32-1.76)* | | 4.06 (3.46-4.76)* | | |
| Women, Black/Brown, Married | 2.26 (1.81-2.82)* | 4.57 (3.35-6.24)* | | 2.81 (2.47-3.21)* | | 5.03 (4.30-5.90)* | | |
| Women, Black/Brown, Single | 2.99 (2.37-3.79)* | 8.40 (6.15-11.47)* | | 2.45 (2.19-2.74)* | | 5.33 (4.60-6.15)* | | |

* Statistical significance (p<0.05). Multinomial Logistic regression. Models adjusted for region, area of the household, resident’s number and income. cRRR: crude estimates of relative risk ratio. ¹PNAD (*Pesquisas Nacionais por Amostras de Domicílios*): Brazilian National Households Sample Surveys; ^2^POF (*Pesquisa de Orçamentos Familiares*): Household Budget Survey.
