# Supplementary material for: Gender, skin color, and household composition explain inequities in household food insecurity in Brazil
Source: PLOS Glob Public Health. 2023 Oct 3;3(10):e0002324. doi: 10.1371/journal.pgph.0002324 (PMC10547153; doi:10.1371/journal.pgph.0002324)
Supplement: Attachment — Submitted filename: Response to Reviewers_Final.docx [file pgph.0002324.s005.docx]

Dr. Catherine Kyobutungi and Madhukar Pai,

Editors-in-Chief

Journal Plos Global Public Health

July 10th, 2023

Dear reviewers,

We hope this letter finds you well. First, we would like to express our sincere gratitude for your time and effort in reviewing the paper titled "**Gender, racial and household composition explain inequities in household food insecurity in Brazil**" submitted to the Plos Global Public Health. Your valuable suggestions and comments have significantly contributed to improving the quality and clarity of the manuscript.

We also carefully reviewed the entire manuscript, improving the quality of figures and tables and changing the reference list. We have included new references to respond to reviewers' suggestions, and removed older references whose content was contemplated in current references already cited.

We assure you that we have carefully considered each of your suggestions and incorporated most of them into the revised version of the paper. Thus, the manuscript has undoubtedly benefited from your expertise, and we are confident that this final version is greatly improved as a result.

We hope that the changes made are in accordance with your expectations. See below the answers for each one.

Sincerely,

Lissandra A Santos

International Center of Equity in Health

lisamorims@gmail.com

Rafael Pérez-Escamilla

Yale School of Public Health

rafael.perez-escamilla@yale.edu

Rosana Salles-Costa

Federal University of Rio de Janeiro

rosana@nutricao.ufrj.br

**ANSWERS TO THE REVIEWER’S COMMENTS**

**(In the responses below, author comments are in red and bold)**

**Reviewer #1**

1. Abstract: Consider opening with 1-2 sentences about the why this study was conducted, and the knowledge gaps it aims to address.
**R: We appreciate the suggestion, change made (L. 3 to 5).**

*“*It is well known that female-headed households (FHHs) are more likely to experience food insecurity (FI) than male-headed households (MHHs), however there is a dearth of evidence on how gender intersects with other social determinants of FI.*”*

2. Abstract: Remove causal language. For instance, replace “increased the risk of” with “was associated with higher risk of”.

**R: We appreciate the suggestion, change made (L.19).**

*“*The presence of children in the household was associated with a higher probability (…)*”*

3. Abstract: If possible, add additional methodological details, particularly information about how households were classified into one of the eight gender-race-marital classes.

**R: We appreciate the suggestion, but there is a word limit in this section. The information is described in the Data section (item 2.3), within the manuscript.**

4. Introduction: To attract a wider readership who may not be familiar with the concept of food insecurity, please add greater detail about this construct. Specifically, it would be helpful to include a definition of food insecurity as well as findings demonstrating its negative impacts on well-being. Such information would help showcase why food insecurity is an important indicator to track and intervene upon for improving public health.

**R: We appreciate the suggestions. The definitions were added (L. 37 to 41).**

“The FAO defines FI as a condition that exists “when people lack secure access to sufficient amounts of safe and nutritious food for normal growth and development and an active and healthy life” [2]. The definition of FI in Brazil also considers the potential psychosocial consequences of this condition, including the feelings of social exclusion, low self-esteem, stress, and emotional suffering [3].”

5. Introduction: The authors cite relevant literature to support the notion that female-headed households have less access to financial capital and thus may be at greater risk of experiencing food insecurity. I think it would also be compelling to note that females may also have lower social capital, experience substantial barriers to accessing and benefiting from affordable foods (e.g., engaging in transactional sex to acquire preferred foods [see Fiorella, K. J. et al. A review of transactional sex for natural resources: Under-researched, overstated, or unique to fishing economies?]), or alter their behaviors to buffer other household members from the negative consequences of food insecurity [see Maxfield, A. Testing the theoretical similarities between food and water insecurity: Buffering hypothesis and effects on mental wellbeing.].

**R: We appreciate the suggestions. We have modified the text accordingly (L. 50 to 54).**

“Women play a central role in household FS due to the household activities that they have historically been responsible for, including caregiving, which involves protecting the health and food security of children and other family members [6-8]. This is challenging for women as they usually have lower social capital, and experience substantial barriers related to gender norms to accessing healthy foods [9, 10].”

6. Introduction: Most of the introduction describes the factors that plausibly increase the risk of food insecurity among female-headed, related to male-headed, households. It would be useful to provide similar explanations for the other characteristics under investigation, namely race/skin color and marital status. The authors should also incorporate relevant theoretical evidence about the importance and development of intersectional theory. I encourage the authors to review a recent paper that provides a concise history of the concept of intersectionality and its utility for public health [Shah, S. H. et al. Variations in household water affordability and water insecurity: An intersectional perspective from 18 low- and middle-income countries.

**R: The suggestion was well noted. We have covered more in depth the construct of intersectionality (L.61-71).**

“Furthermore, gender intersects with other social determinants of health compounding women’s social vulnerabilities. For example, the intersection of female gender with black/brown race and marital status, increases substantially the risk of FI. It is well known that households headed by indigenous and black people around the world, are more likely to report FI when compared to households headed by whites or the dominant race [12, 14, 16, 18]. It is also well known that the lifetime accumulation of social and economic disadvantage among black people is associated with a substantial increase in the risk of FI within this population [19]. Finally, non-white women rearing children or adolescents as single mothers are more likely to report FI [20] and have worse living conditions than those living in households with other family structures, regardless of gender [21].”

7. Introduction: The authors clearly state why patterns of food insecurity should be examined in Latin American and Caribbean countries but do not justify why Brazil should be studied in particular. It may be helpful to include empirical evidence that food insecurity is a prevalent issue in Brazil and demonstrated to undermine health and well-being.

**R: Thanks for your highly relevant suggestion that will benefit a lot the journal’s international readership. A brief historical background about household food insecurity time trends in Brazil has been added (L. 42-49).**

In the early 2000s, Brazilian households were experiencing major FI challenges and this situation motivated a series of social movements that culminated with the development of the Brazilian Food Insecurity Scale [*Escala Brasileira de Insegurança Alimentar* (EBIA)] in 2004 [3,4]. Between 2004 and until 2013, there were substantial reductions in FI rates in Brazil but since 2016 this trend reversed. Household FI worsened dramatically as a result of a political and economic crisis that strongly undermined the food security (FS) policies that were successfully implemented during the previous political administrations [5].*”*

8. Methods: Were there any additional inclusion/exclusion criteria? For instance, what was the minimum age to participate?

**R: For PNAD and POF surveys, the interviewer is instructed not to accept a person under 14 years of age as an informant. Usually, the household reference person is the one sought to answer the questions, but in their absence, another person can respond (L. 144-147).**

*“*All survey participants who responded to EBIA on behalf of the household (usually the heads of household) were included in our analyses. However, it is important to note that, for both surveys, the interviewers were instructed not to accept a person under 14 years of age as an informant.*”*

9. Methods: Were all surveys conducted during the same time of year/season? Could variations in the timing of surveys account for some of the differences observed between survey waves?
**R: We appreciate the suggestion, change made (L.123 to 126).**

*“*PNAD surveys are applied during three months of the year, while POF is applied during the whole year, but access to food in Brazil is more influenced by income, rather than seasonal variations in food intake [31, 32], hence this is unlikely to have affected the comparability across surveys.*”*

10. Methods: What language(s) were surveys conducted in? Were any of the survey modules piloted before broad implementation to ensure that items were understood as intended?

**R: In Brazil, the vast majority of the population speaks Portuguese, and this is the population included in the surveys. For this reason, all people were interviewed in Portuguese. In Brazil there are specific surveys conducted only among indigenous people who speak their traditional languages. Regarding the pilot modules, there are pilot-tests carried out by IBGE before each survey. We are now directing to the references more they can find more methodological details (L. 142 and 143).**

“More specific details on the surveys’ methods and procedures can be found elsewhere [29, 33, 34].”

11. Methods: Describe how food insecurity scores were scaled to ensure comparability between households with and without children.

**R: The EBIA is used for both households, with and without children. The difference is made in the total number of questions, of which 14 are for families with children and/or adolescents (<18 y of age), and 8 items referring to adult-only households. This difference is also considered in the classification score of the families in the levels of FI. Details have been included in the text as shown below. The psychometric validity of this approach has been extensively documented (see L. 174 to 183).**

*“*The current version of the EBIA consists of 14 questions with dichotomous answer options (“yes” or “no”). Eight items apply only to adults in the households (aged 18 years or above), and 6 items apply exclusively to households with children and/or adolescents (aged 18 years or less). Each household is assigned an additive score based on the total number of EBIA items affirmed. The score is then coded into three categories based on standard cutoff points to classify each household as food secure (0 point with or without people under 18 years of age), mildly FI (only adults: 1-3 points; with children: 1-5 points), moderately FI (only adults: 4-5 points; with children: 6-9 points), or severely FI (only adults: 6-8 points; with children: 10-14 points) [39].*”*

12. Methods: It would be helpful to provide the theoretical range of food insecurity scores as well as the cut-points for determining different levels of food insecurity.

**R: We appreciate the suggestion, change made (see above).**

13. Methods: Consider reporting the internal validity of the food insecurity scale across sampling waves.

**R: We are now reporting EBIA’s Cronbach’s alpha at each wave and in all instances, it showed excellent internal validity. We are also encouraging the readers to read the extensive prior validation work of EBIA (see L. 155 to 161).**

*“*Household FI, the primary outcome in our analyses, was measured with the Brazilian Household Food Insecurity Measurement Scale (*Escala Brasileira de Insegurança Alimentar* - EBIA), which has been repeatedly validated since 2004 for its construct, face, internal consistency, predictive and concurrent validity (Cronbach's alpha ranged from 0.91 to 0.94). As a result, EBIA is considered to be a well validated robust experience-based scale to assess household FI in rural and urban households in Brazil [4, 35, 36].”

14. Methods: What proportion of respondents had missing data? How were missing data handled? Either describe how missing data are unlikely to meaningfully influence results or conduct sensitivity analyses to estimate the potential bias introduced from complete-case analysis.

**R: We appreciate the suggestion. We clarified this topic in the main text (L.144 to 152).**

“All survey participants who responded to EBIA on behalf of the household (usually the heads of household) were included in our analyses. However, it is important to note that, for both surveys, the interviewers were instructed not to accept a person under 14 years of age as an informant. Furthermore, households headed by individuals who self-identified as being “yellow” and indigenous were excluded because of their lack of representativeness in the survey (0.6% in 2004, 0.9% in 2013 and 1.2% in 2018). Among those responding to EBIA a very small percent had missing information in any of the variables of interest (1.4% in 2004;3.7% in 2013; no missing data in 2018), hence missing data was not an issue in our study.”

15. Methods: Is there empirical evidence to support that the findings from the two different surveys are comparable? Additional justification is needed given that this is a critical assumption of the study.

**R: The two types of surveys have a common nationally representative sampling framework. In addition, the use of the previously validated EBIA in both surveys to assess FI allowed for direct comparability of the primary outcome across surveys (L. 123 to 130 and 136 to 143).**

“PNAD surveys are applied during three months of the year, while POF is applied during the whole year, but access to food in Brazil is more influenced by income, rather than seasonal variations in food intake [31, 32], hence this is unlikely to have affected the comparability across surveys. Even though the two surveys used slightly different approaches to household selection, the common sampling framework and just focusing on permanent private household ensured that comparability between both surveys, which are representative at the national and macroregional levels as well as urban and rural areas.

(…)

The PNAD surveys are applied to different types of households, including permanent private households and collective living households, such as hotels, pensions, orphanages, or asylums. By contrast, the POF survey only included permanent private households. To ensure the comparability between POF and PNAD surveys, in the case of PNAD we selected only the permanent private households for the analyses. Thus, in our analyses both surveys are comparable in terms of sampling design and meth­odology to estimate food security and FI in permanent private households. More specific details on the surveys’ methods and procedures can be found elsewhere [29, 33, 34].”

16. Methods: What proportion of individuals identified as having black compared to brown skin? What is the justification for combining these two categories?

**R: Thanks for your query. These data are provided in the subsection 2.3 (L.206-215).**

*“*The proportion of people who self-identified as black (based on skin color) ranged between 7.5% and 11.4% across surveys, and the proportion of people who considered themselves to be brown (*pardos,* in Portuguese) ranged between 42.7% and 49.3%. Both racial categories were combined because Brazilian race dynamics are “ambiguous and fluid” and the boundaries between black and *pardos* are blurry as both are Afro-descendant and experience systematic racism and discrimination [41]. Furthermore, the Brazilian black movement highlights the political importance of representing the Afrodescendant population as the people self-identifying as black or brown based on their skin color [41, 42]. Thus, for this study, the skin color variable was further reclassified into two categories (white or black/brown).*”*

17. Methods: How was urban/rural household location determined?

**R:** **Urban areas were defined as areas corresponding to cities (municipal seat), towns (district seat) or isolated urban areas with more than 50,000 inhabitants in a densely populated area while rural areas were comprised as areas of dense occupation with no more than 3,000 inhabitants, or the rest of the areas outside the aforementioned urban areas. As indicated in manuscript, both surveys are representative of the urban and rural populations of Brazil (L.131-135).**

*“*The IBGE surveys classify the area of residence as urban or rural the same way as the Brazilian demographic census does. Urban areas were defined as areas corresponding to cities (municipal seat), towns (district seat) or isolated urban areas with more than 50,000 inhabitants in a densely populated area. By contrast, rural areas were those towns with no more than 3,000 inhabitants, or those outside the urban areas [32].*”*

18. Methods: When determining which confounders to include in the model, why was a data-driven, as opposed to a theoretically driven, approach used? As noted in numerous epidemiological reports, such approaches can introduce bias [e.g., VanderWeele, T. J. Principles of confounder selection]. Consider running several models as a sensitivity analysis to evaluate whether findings are consistent across model specification.

**R: Thanks for pointing this key issue out. In fact, theoretical considerations were also taken heavily into account in the selection of confounders. Specifically, the framing of this study is based upon the doctoral thesis from the first author of this article that contains the theoretical model used for confounder determination, as well as the model proposed by Kepple and Segall-Correa (2011), as described in the manuscript (see lines 226 to 232).**

19. Methods: What information led the authors to conclude that having children less than 5 in the household was an effect measure modifier? Please include these details in the main text of the manuscript.

**R: The interaction was tested with Stata software. We add this information to subsection 2.5 (see L. 250 and 251).**

*“*Effect modification by the presence of children under 5 years of age in the household was examined through the testing of statistical interactions.*”*

20. Methods: The authors note that “multinomial logistic regressions were conducted for each of the three surveys to estimate the prevalence of mild and moderate/severe FI across the intersection of gender”, but then report odds ratios. Is it possible to fit generalized linear models to obtain prevalence differences between groups, which are more valuable for informing public health decisions than relative measures [see Poole, C. On the Origin of Risk Relativism]?

**R: We appreciate the query. After careful consideration, we concluded that the most appropriate estimate to use is the relative risk ratio (RRR) which is generated by the multinomial logistic regression. The changes have been made to the corresponding graphs and figures. Thanks for suggesting the reference by Poole (2010). After carefully considering it, we decided to use a relative estimate because the author proposes the adoption of absolute measures in the context of causality interpretation, which does not apply well to our study based on cross-sectional analyses.**

21. Methods: Please describe how informed consent and ethical approvals were obtained within the parent studies.

**R: Thank you for the query. We add this information to the manuscript (see L. 264-272).**

*“*IBGE's activities and surveys are governed by Law N^o^ 5,534, of November 14, 1968, that requires the government to provide access to the public to the survey data and information. According to this Law, all individuals and legal entities are guaranteed confidentiality while at the same time requiring them to answer the surveys administrated by IBGE, with reassurances that all information provided will be used exclusively for deidentified statistical analysis purposes. Additionally, the secondary data analyses presented in this article are based on public domain information and did not require additional IRB approval according to Resolution N^o^ 510 of April 07, 2016, from the National Committee of Ethics in Research (CONEP).*”*

22. Methods: The use of multinomial logistic regression is sensible. I wonder, however, if ordinal logistic regression was considered given that the outcome is ordinal.

**R: We carefully considered this point a priori because the use of either ordinal or multinomial logistic regressions would have been appropriate to analyze our data. Even though the ordinal logistic regression could have increased the statistical power, the fact that this model estimates “an average effect of the predictor across all outcome levels”, could have led our study to miss the different effects between the outcome levels (Sainani, 2021). For this reason and the fact that multinomial logistic regression can be applied to either ordered or unordered outcomes we decided to use this statistical modeling approach.**

**Reference**

**1) Sainani KL. Multinomial and ordinal logistic regression. PM R. 2021 Sep;13(9):1050-1055. doi: 10.1002/pmrj.12622. Epub 2021 Jun 14. PMID: 33905601.**

23. Methods: Note why married white men were selected as the referent category.

**R: We appreciate the suggestion. We added this information (L. 251 to 253).**

“Married white men had the lowest prevalence of moderate/severe FI and one of the highest prevalence of FS in all survey years, this is why it is considered the reference profile.”

24. Results: In Figure 1, please consider including error bars around the point estimates.

**R: We appreciate the suggestion. Done.**

25. Results: In Table 1, “qui-squared” should be “chi-squared”. Also, make clear in the caption that gender refers to that of the household head, not necessarily that of the survey respondent.

**R: We appreciate the suggestions. Recommended edit made on the Table 1. Additionally, we clarified that we use the expressions ‘head of household’ and ‘reference person’ as synonymous, as described in the definition of terms (L 188-191). We added clarification about the survey respondent characteristics in lines 191 to 193.**

It is important to note that usually the survey respondent was the reference person of the household, but when they were absent the oldest person in the household was interviewed [29, 33, 34].”

26. Results: Consider also presenting aggregate food insecurity data across time (i.e., not divided by subgroups) to understand population-level trends.

**R: We appreciate the suggestion. Done, please see Figure 1.**

27. Results: I am finding it difficult to interpret Figure 2. Please consider providing more information in the legend to make clear what each bar represents. For instance, one bar is meant to represent “gender and race”, but which gender and race category?

**R: We appreciate the suggestion. We created a new graph specifying the profiles used for each one of the dimensions, and we added more interpretation to the main text (L. 315 to 325).**

*“*Figure 2 shows how the stepwise addition of the household head characteristics gender, race/skin color, marital status, and the presence of children in households led to a stepwise increase in the prevalence of moderate/severe FI in households (numbers are provided on TableS2).

Compared to the 'white married’ profile (first bar in Figure 2), women had higher FI prevalence across survey years. When the heads of the household declared themselves to be black or brown (based on skin color) FI prevalence increased even more. Following a stepwise trend, FI increased after adding that the household was single headed, and then when adding the presence of children younger than 5 years old in the household.

Importantly, the increase in percentage points by profile differed between men and women, and over time.*”*

28. Results: When comparing changes across time, it might be useful to include estimated percentage-point changes to support statements like “men had the greatest increases across time”.
**R: We appreciate the suggestion. We have made the changes (L. 328 to 331).**

*“*In 2013, the increase in these households were of 1.3 percentual points (pp.) and of 0.8 pp. in 2018. In 2004, in households headed by single black/brown women who had children less than 5 years old, the prevalence of moderate/severe FI was 16.7 pp. higher than for households headed by married white women.”

29. Results: At line 246, would it be more accurate to replace “likelihood of FI” with “prevalence of FI”?

**R: We appreciate the suggestion;the sentence have changed (line 343-346).**

30. Results: Table 2 includes a lot of great information, but it is difficult to interpret. Consider creating a coefficient plot that includes point estimates for each “person type” across the years. This would be useful to track temporal trends and highlight which groups are consistently at greatest risk for experiencing food insecurity.

**R: We appreciate the suggestion. We did a new graphic representation of these data and displayed the crude estimates on a new supplementary table. Please see Figure 3 and S4 Table.**

31. Results: Please provide information about the percent of households categorized into each of the eight categories across time (e.g., what percent of households were headed by white single men at sampling period?). Currently, such information cannot be derived from Table 1.

**R: We appreciate the suggestion. We are adding this information as supplemental material. Please see S3 Table file.**

32. Discussion: The Discussion is strong and engages deeply with relevant literature. I only have a few suggestions on how to potentially increase its impact. For instance, it may be helpful to describe why there were such dramatic shifts in the percentage of households headed by females across sampling waves. Is there evidence of demographic shifts or changes in gender norms across the 14-year study period? Did the surveys include probes for soliciting information about the gender of the household head that potentially changed across survey waves?

**R: Thanks for your kind comment. We appreciate the suggestions. We added some key information about this trend (L. 411 to 423).**

“In recent years, there has been a significant increase in the percentage of female-headed households in several countries around the world, in which, Latin America and the Caribbean represent the region with the highest proportion of households headed by women (36.2%) [21, 47]. In Brazil, this trend has also been reported with the percentage of female household headship increasing gradually over time reaching almost half of Brazilian households nowadays [48, 49]. Changes in cultural patterns, such as the decline of marriage, the increase in consensual unions, and a greater number of divorce or separation, in addition to the greater female participation in the labor market and women's higher education are factors likely explaining some of the trend in the increase in FHHs prevalence [21, 47-51]. Changes to the question to identify the head of household could also explain the growth of female headship reported, however the questions about the head of the household were asked the same way to the respondents across surveys and across time.”

33. Discussion: At line 304, consider listing the specific policy and programmatic changes that were made. For instance, was support for and benefits included in all social programs reduced, or specifically those related to nutrition?

**R: Even though we think this is a great observation, considering the recommendation of the journal of “to present and discuss the findings concisely” and being aware of the scope of this manuscript (engaging on policy and programmatic implications could lead to a whole new paper), we have decided to point the reader to the reference where they can very detailed information on this topic.**

34. Discussion: Provide more details about the studies described and cited at line 307. Which regions of the world were these studies conducted in?

**R: We appreciate the suggestion. We have added regions where the studies were conducted (L. 424 and 426).**

“Consistent with our findings, previous studies conducted in African, Asian, and Latin American countries have shown a higher risk of being poor and reporting FI in FHHs than in MHHs [12, 15, 52-55].”

35. Discussion: Consider describing the utility of measuring individual food insecurity in future studies to understand intra-household variations in food insecurity experiences.

**R: We appreciate the suggestion. We added the information at the end of the Conclusions section (L. 572 to 576).**

“We also encourage further studies focused on exploring other ways of assessing food insecurity issues, such as measures that identify if and how different members of the same household experience FI to understand how to ensure that FS policies address the needs of the most vulnerable within the households, including, women, black/brown people, and young children.”

**Reviewer #2**

1. A first limitation of the study resides in the data that dates back to 2018. Since the global outlook of food insecurity has been affected by notable changes in recent years, it would be interesting to refer the analysis to more recent data, or at least to explain to the readers why this has not been done. However, the study has been able to analyse food insecurity in Brazil over a 14-year period with highly comparable nationally representative surveys.

**R: We appreciate the suggestion. Data from 2018 was the last nationally representative survey in Brazil and represents an important pre-pandemic scenario, as now described in lines added to the main text (L. 90 to 96).**

*“*The 2004-2018 period was chosen because of the availability of national representative surveys that assessed FI using EBIA. Since the COVID-19 pandemic exploded in early 2020 Brazil, experienced an additional worsening of FI leading to a sharp increase in the number of hungry people. Hence, the analytical methodology developed for this study to understand FI intersectionality during the pre-pandemic scenario can then be replicated in the context of the pandemic once nationally representative data becomes available from Brazil and other countries.*”*

2. The manuscript can be better organized in the following way:

- Section 2 should be named “Data” rather than “methods”, since it deals with the data used in the study

- Section 2.5 could be numbered “3” as it refers to the empirical methodology

- Section 2.6 can be put as a footnote in section 2.1

**R: We appreciate the suggestions. We changed the name of the section ‘Methods’ for ‘Data’, as suggested. Regarding the other suggestions, we didn't make any changes because: (i) the journal does not allow footnotes and (ii) section 2.5 refers to the analysis of the data, so we think that the subsection still belongs to the Data section.**

3. I should invite the authors to put particular attention to the labels about “race”. I suggest to avoid overlapping the concept of race with that of skin colour without explanation, since this is not universally accepted. I think the authors could write only “race” specifying (starting from the abstract) that they identify “race” with the self-stated colour of the skin (as specified in Section 2.3). More in detail, I suggest to specify the first time the authors use the term “race” that they actually mean the “self-identified phenotype” (both in the abstract and in the introduction). I also think that the label “mixed-race” is misleading, since it refers to brown/black phenotypes. I suggest to find a more accurate label.

**R: Thanks for pointing this key issue out. As mentioned, the manuscript contains information on the operational definition of the term race in Brazil (L 203-204), and we are clarifying at different points in manuscript that it is based on self-reported skin color. However, we agree that the denomination mixed-race is not accurate since it applies to the brown people, but not to the black people. Therefore, we chose to change this label to ‘black/brown’ (L. 212-215).**

“Furthermore, the Brazilian black movement highlights the political importance of representing the Afrodescendant population as the people self-identifying as black or brown based on their skin color [41, 42]. Thus, for this study, the skin color variable was further reclassified into two categories (white or black/brown)”.
